# The impact of social distancing and self-isolation in the last corona COVID-19 outbreak on the body weight in Sulaimani governorate- Kurdistan/Iraq, a prospective case series study

**DOI:** 10.1016/j.amsu.2020.09.024

**Published:** 2020-09-18

**Authors:** Hiwa Omer Ahmed

**Affiliations:** Professor & Senior Lecturer in College of Medicine, University of Sulaimani, Kurdistan region, Iraq

**Keywords:** Coronavirus disease, COVID-19, Obesity, Self-isolation, Social distancing, Stress eating, Weight gain

## Abstract

**Background:**

Our area, corona (COVID-19) quarantine was applied from March 14 to April 23, 2020. It was in all forms, including curfew, social distancing, self-quarantine, area quarantine, self-monitoring, and isolation. Due to their concerns about their safety and families, friends, colleagues, and society, everybody was feeling upset, fearful, and anxious. Several studies have reported an association between prolonged sitting time and weight gain. As a way to relieve the tension during that period, people started stocking up on unhealthy foods like over-salted snacks and over-sweetened sugars. People stayed at home, feeling bored, anxious, and stressed and sought relief by eating. Also, there was a rise in emotional eating when the stress of isolation increased by the closure of gyms, casinos, and movie theaters. Moreover, restaurants were allowed to serve the only takeout. Besides, unemployment was skyrocketing, hospitals were overflowing (or were preparing for the possibility), many people were struggling to meet their basic needs, and no one knows when or how it would end.

**Objective:**

The study aimed to figure out whether social isolation during the COVID-19 quarantine is consistent and associated with emotional eating and gaining weight or not.

**Methods:**

A prospective cross-sectional case series study was conducted on 765 patients who have visited the bariatric clinic in Sulaimani city, Kurdistan-Iraq, from April 23 to June 23, 2020. An individual face-to-face interview was conducted with each participant to obtain signed informed consent, provide them with information about the type and the subject of the work, and ask them the study's questions. Each interview lasted between 30 and 45 min and was conducted in a closed session by two Kurdistan Board trainees.

**Results:**

No patient who was quarantined for the COVID-19 was included in the work, but all other patients were included. The selected patients were those who had undergone social distancing (n = 568, 82.48%) by the local law and did self-isolation (n = 134, 17.51%) at home for reasons like having comorbidity, being prone to contamination due to their jobs (health, police, and media workers), having some family members at home with comorbidity (n = 23, 03.00%), and having comorbidity and having undergone social distancing because of their other family members’ need to do so for their physical disability (n = 25, 03.27%). Almost all patients (n = 741, 96.86%), **even,** those with comorbidity (n = 136, 17.78%)**,** were emotionally stable before the outbreak**.** Seventy-three female patients (09.54%) and138 males (20.65%) sustain their weight during and two months after the outbreak, while the rest (n = 554, 72.41%) gained different amounts of weight.

**Conclusion:**

Social distancing and self-isolation in the last COVID-19 outbreak influenced weight gain, but weight gain of less than 2 kg was observed among almost all patients who gained weight (98.05%). The patients who were gain more than 3 kg were mostly females or/and from the center of large cities.

## Highlights

• There is an association between prolonged sitting time and weight gain.
• In the outbreak of corona COVID-19, immediate challenges impacted our personal relationships.
• Emotional eating escalated more when the stress of isolation increased.
• Bored isolated peoples in this social distancing, bombarding audience by unscientific data over social media.
• The impact of the quarantine is continuous after the period was ended.

## 1 Introduction

The word quarantine was first used in Venice, Italy, in 1127 regarding leprosy and was widely used in response to the Black Death. It was not until 300 years later that the UK properly began to impose quarantine in response to the plague. Most recently, quarantine was used in the coronavirus disease 2019 (COVID-19) outbreak [1].

It is an unprecedented challenge with immediate impacts on our relations which, have radically changed as we have learned distance ourselves socially, wear face masks while walking or shopping, smile more with our eyes, and nod or waves our greetings [2].

It is a fact that social distancing can reduce virus transmission [3]. On the other side, however, separation from loved ones, the loss of freedom, uncertainty about disease status, and boredom can, on occasion, create dramatic effects. There have been reports on suicide attempts after substantial anxiety generated by COVID-19, and lawsuits were brought following the imposition of quarantine in previous outbreaks [1,2,and4]] which, could lead to feelings and memories of previous traumatic events and the distress that we feel [5,6].

From March 14 to April 23, 2020, in our area, corona (COVID-19) quarantine was applied in all forms, including curfew, social distancing, self-quarantine, area quarantine, self-monitoring, and isolation. During the quarantine, all people were feeling upset, fearful, and anxious about their personal safety and that of their family, friends, colleagues, and society [5].

Several studies have reported an association between prolonged sitting time and weight gain [7,8]. People try to relieve their tension by stocking up on unhealthy foods like over-salted snacks and over-sweetened sugars during quarantines [9,10]. They stay at home all the time and get bored, anxious, and stressed, so they seek relief by eating all the day [11]. Moreover, emotional eating escalates more when the stress of isolation is increased due to the closure of gyms, casinos, and movie theaters, and those virtual restaurants are only allowed to serve takeout [9,12]. Besides, unemployment is skyrocketing, hospitals are overflowing, many people struggle to meet their basic needs, and no one knows when or how this will all end [13]. In these stressful situations, most people become bored, and when one becomes bored, he/she is quite likely to eat more [9]. Everybody is terrified of weight gain even amid an unprecedented global pandemic because they are constantly flooded with messages showing the very bad effects of gaining weight or living in a larger body [13]. Gaining weight is, in fact, growing risk to the health of people in developed nations and has been described as an epidemic that has become a global health concern [14].

Another cofactor in increasing boredom and stressful, emotional eating is the uncensored media health-wise, which may be negatively associated with health-related outcomes [15]. Especially on bored isolated peoples in social distancing, bombarding the audience by unscientific data like “I think that in these times, it is not a time to diet and to make yourself crazy.^”^ [11] TikTok videos, memes, stories, essays, and poems about living in isolation all become part of the culture [15,16]. In another study, social media use is conceptualized as normal social behavior [15]. Some go extreme and say that we do not need to shame ourselves for wanting treats and gaining weight. We are trying to survive an unprecedented global situation. Indeed, that is task enough right now? Wrote Tracy Isaacs, a Toronto professor and co-author of “Fit at Mid-Life.” [17] Underneath the memes is the unspoken assumption that the pandemic will automatically lead to weight gain for everyone, which is not logical at all [13].

Also, O'Malley pointed out that if you allow yourself to eat what you want when you want guiltlessly, you likely feel more in control around food and be able to stop eating when you are full. ^13)^ On the other hand, obsession with weight gain and setting rules around what you eat can perpetuate that out-of-control feeling [13,17,18]. However, it may provide some comfort to know that thousands of other people are going through the same thing [19].

Quarantine may increase overeating in different ways. Stress experiences can be emotional challenges (such as interpersonal conflict, loss of loved ones, and unemployment) or physiological challenges (such as food deprivation, illness, and drug withdrawal states) [20]. Also, Dr. Dariush Mozaffarian, people buy shelf-stable foods that do not require much time to prepare [11]. Moreover, all gyms are closed [21]^,^ which is a disappointment for their regular customers [22]. In addition, binge-shopping may become a way of life [9], and people tend to fall back on lazy patterns, letting the vigilance with the food go, which is comfort food – it is feel-good food and keeps them full [11].

Moreover, O'Malley pointed out that the idea that people are eating different foods and in a different pattern than before makes sense because when things change, other things consequently change [13]. Hyper palatable food (e.g., high-fat, high sugar) may possess addictive qualities, and stress is an essential factor in addiction development, which may also contribute to an increased risk of obesity and other metabolic diseases [18]. Being stuck in a home with only Netflix and comfort snacks in the middle of what is arguably the most uncertain time in modern history, many people were more worried about getting fat than contracting an illness that makes it so difficult to breathe that might cause death [23].

Emotional eating or overeating in response to negative emotions is a behavior endorsed by both normal and overweight/obese people. For some individuals, emotional eating contributes to weight gain and difficulties losing weight [24] and association with overweight and obesity concerns. [25].

Some have an opposite view on the effect of the new situation in this outbreak, suggesting that the food prepared outside the home is generally less healthy than home-cooked meals, so there is an optimistic view that the break from restaurant eating could be positive when it comes to nutrition causing people to be healthier than they were before because they are not eating at restaurants [4].

The present study was conducted to answer this question. Is social isolation during the corona COVID -19 quarantine consistent and equally associated with emotional eating and weight gain?

## 2 Patients, materials, and methods

In line with The STROCSS Guideline [26], a prospective cross-sectional case series study was conducted on 765 patients who had visited one of the bariatric clinics in Sulaimani city, Kurdistan-Iraq from April 23 to June 23, 2020. Through individual face-to- face interviews, signed informed consent was obtained from each of the patients, the type and the subject of the work were explained to them, and they were asked the study's questions. The duration of each interview was between 30 and 45 min, and they were conducted in a closed session by two Kurdistan Board trainees.

Necessary approval was obtained from the Ethical Committee of Sulaimani University-College of Medicine. Moreover, informed consent was taken from each patient during the face-to- face interviews. Part of the questionnaire was a modified SF-36 questionnaire [27] for health to assess patient's perceived satisfaction or dissatisfaction in the significant domains of including (life stress, boredom, loneliness, depression, and suicidal thoughts). Each item has five tail questions, to select one of the five (not at all, a little bit, moderately, quite a bit, or extremely).

The rest of the questionnaire included questions examining three aspects of the dilemma: type of social distancing, how to label their emotional state, and any changes they made in their meals and type of meals. Finally, all of the patients were weighed by a digital scale, and a stadiometer measured their height. Their measured weights and heights were compared with self-reported weights and heights. To avoid bias in reading their scales and the digital scale, they were also asked to show their pictures before the outbreak to imagine the changes in their weight. The majority of participants expressed concerns about eventual weight gain. The statistical analysis was performed with Statistical Package for the Social Sciences (SPSS) version 21. Chi-square test adjusted for clinical characteristics was assessed at the conventional 0.05 level of significance, considering any p-value ≤ 0.05 as statistically significant.

## 3 Results

The study did not include any patients who were quarantined for COVID-19, but it included all other patients who underwent social distancing (n = 568, 82.48%) by the local law, did self-isolation (n = 134, 17.51%) at home for reasons like having comorbidity or being prone to contamination due to their jobs (health, police, and media workers). Those having some family member at home with comorbidity (n = 3, 03.00%); and had comorbidity and underwent social distancing because of their other family members need to do so because of their physical disability.

The most common age group was 21–30 years, with 349 patients (45.62%), followed by 31–40 years consisting of 208 patients (27.18%). The smallest age groups were 70 years and more with ten patients (01.31%) and those under the age of 20 years, with nine patients (01.17%).

About two-thirds of the patients were male (n = 463, 60.53%), and the male to female ratio was 1:0.65. Most of the patients were from the Sulaimani governorate center (n = 83, 50.11%), some from rural areas (n = 231, 30.19%), a few small cities (n = 140, 18.30%), and a minority (n = 11, 01.44%) from Europe and other countries who had just arrived in the area before the outbreak to visit their families and friends (See Table 1).

**Table 1:** Demographic data about the participants.

Variables			Number and frequency (n = 765)
**Types of distancing**	Quarantined		Zero
	**Social distancing**	**healthy**	606 (79.21%)
		**Comorbid**	025 (03.27%)
	**Self-isolation**	**healthy**	023 (03.01%)
		**Comorbid**	111 (14.50%)
**Age**	**Below 20 years**		009 (01.17%)
	**21**–**30**		349 (45.62%)
	**31**–**40**		208 (27.18%)
	**41**–**50**		120 (15.68%)
	**51**–**60**		039 (05.09%)
	**61**–**70**		030 (03.92%)
	**Above 70**		010 (01.31)
**Gender**	**♀**		302 (39.47%)
	**♂**		463 (60.53%)
**Residency**	**The center**		383 (50.11%)
	**Small city**		140 (18.30%)
	**Rural area**		231 (30.19%)
	**Europe and other countries**		011 (01.44%)

Almost all patients (n = 741, 96.86%) and even those with comorbidity (n = 136, 17.78%) were emotionally stable before the outbreak. However, about one-third of them became emotionally unstable during the outbreak (n = 521, 68.11%) and after freedom from quarantine (n = 509, 66.53%). The instability was in the form of feeling overwhelmingly stressed during (n = 231, 30.19%) and after freedom from the situation (n = 267, 34.90%). It is noticeable that even after the isolation process calmed down, the stress was present in more patients compared to the period of the outbreak. One hundred forty-five patients (18.95%) labeled themselves as bored during the outbreak, and a smaller number of them (n = 76, 09.93%) felt bored after the outbreak.

The same was true for the feeling of loneliness with (n = 131, 17.12%) and (n = 15, 01.96%) during and after the quarantine, respectively. Moreover, depression was more after the lockdown was lifted (n-148, 19.34%) in comparison with the period of staying at home (n = 13, 01.69%). The same was true regarding suicidal thoughts, such that none of the patients had this impulse before, while one patient (00.13%) developed it during the lockdown, and the numbers escalate to three patients (00.39%) after release from the quarantine (See Table 2).

**Table 2:** Emotional state of the patients before, during and after the outbreak.

Variables	Before the out break	During the out break	after the out break	P-value
**Stable**	741 (96.86%)	244 (31.89%)	256 (33.46%)	0.00391
**Stressed**	13 (01.69%)	231 (30.19%)	267 (34.90%)	
**Bored**	2 (00.26%)	145 (18.95%)	76 (09.93%)	
**Loneliness**	4 (00.52%)	131 (17.12%)	15 (01.96%)	
**Depressed**	5 (00.65%)	13 (01.69%)	148 (19.34%)	
**Suicidal thoughts**	0 (00.00%)	1 (00.13%)	3 (00.39%)	

Slightly more than one-third of the patients, including 73 females (09.54%) and 138 males (20.65%), sustained their weight after the outbreak, while the rest (n = 554, 72.41%) gained different amounts of weight. More than half of the patients (n = 410. 53.59) gained less than 1 kg, of whom 171 were females (22.35%), and 239 were males (31.24%). Also, 129 patients gained 1–2 kg, while only 12 patients (01.97%) gained 3–5 kg. Moreover, two females and one male gained more than 5 kg (See Table 3, Table 4, and Flowchart 1), showing the changes in each group's BMI throughout the blockade.

**Flow chart 1:**
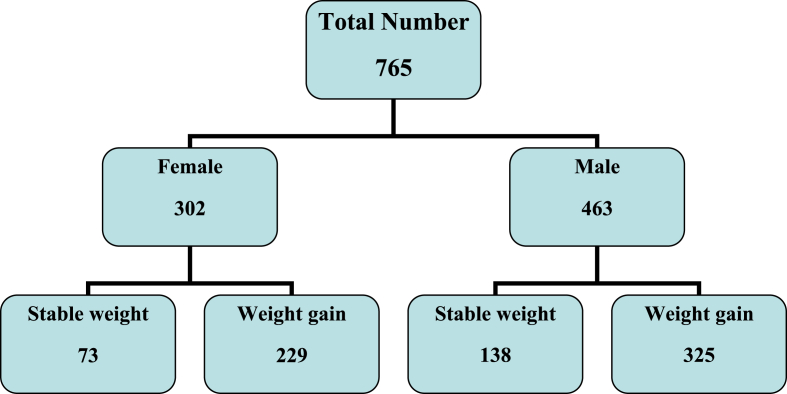
Showing frequency and percentage of the patients with stable or gaining weight in each gender.

**Table 3:** Change in BMI in both genders after the release from blockade.

Variables	♀ (n = 302)<				♂ (n = 463)					P-value
	Median of BMI Before the out break		Median of new BMI on evaluation		Median of BMI Before the out break		Median of new BMI on evaluation			
	Number	BMI (median)	Number	BMI (median)	Number	BMI (median)		Number	BMI (median)	
**Stable weight**	73 **(09.54%)**	24.61	73 **(09.54%)**	24.61	138 (20.65%)	**24.90**		138 (20.65%)	**24**–**90**	0.00547
**Wight change**	171 **(22.35%)**	**23.88**	171 **(22.35%)**	**24.24**	239 **(31.24%)**	25.26		239 **(31.24%)**	25.61	
	48 **(06.27%)**	23.44	48 **(06.27%)**	24.22	81 **(10.58%)**	24.22		81 **(10.58%)**	24.91	
	8 **(01.45%)**	23.15	8 **(01.45%)**	24.69	4 **(00.52%)**	23.88		4 **(00.52%)**	25.26	
	2 **(00.26%)**	25.64	2 **(00.26%)**	28,04	1 **(00.13%)**	24.57		1 **(00.13%)**	26.64

**Table 4:** Change in weight, and BMI in both genders after the release from blockade.

Variables		♀ Weight Kg	♀ Increased BMI	♂ (n = 463)	♂ Increased BMI	P-value
**Stable weight**		73 (09.54%)	000 kg/m^2^	138 (20.65%)	000 kg/m^2^	0.00541
**Wight change**	**>1 kg**	171 **(22.35%)**	0.36 kg/m^2^	239 **(31.24%)**	0.35 kg/m^2^	
	**1**–**2 kg**	48 **(06.27%)**	0.78 kg/m^2^	81 **(10.58%)**	0.69 kg/m^2^	
	**3**–**5 kg**	8 **(01.45%)**	1.54 kg/m^2^	4 **(00.52%)**	1.38 kg/m^2^	
	**> 5 kg**	2 **(00.26%)**	2.40 kg/m^2^	1 **(00.13%)**	2.07 kg/m^2^	

## 4 Discussion

The present study aimed to find the impact of social isolation during the COVID-19 quarantine on gaining weight.

In our area, the COVID-19 quarantine started on March 14 and was lifted on April 23, 2020. It was applied in all forms, including curfew, social distancing, self-quarantine, area quarantine, self-monitoring, and isolation.

### 4.1 Type of the process

There was no chance to contact and include any patient quarantined for COVID-19, so they were not included in the study. See Table 1.

a. Of the total 765 included patients, 568 (82.48%) underwent social distancing by the local law, while a number of those with comorbidity were not suitable for self-isolation, so they were obligated to be in contact with a member of their families because of their physical disability.
b. One hundred thirty-four patients (17.51%) were in self-isolation at home, either because they had comorbidity or were at risk of potential contamination because of their careers (health, police, and media workers), to protect a family member at home with comorbidity (n = 23, 03.00%).

All prevention measures utilized for the communicable COVID-19 disease cause loss of usual routine and reduce social and physical contact with others, which have frequently been reported to cause boredom, frustration, and a sense of isolation from the rest of the world which cause the participants to experience distress, depression, stress, low mood, irritability, insomnia, and post-traumatic stress symptoms [28]. This frustration is exacerbated by not being able to take part in usual day-to-day activities, such as shopping for necessities [1], which has been associated with harmful health outcomes [29]. There is significant uncertainty; how exactly one will stay on track with his weight loss goals might not be at the forefront of the mind [30].

### 4.2 Emotional status

The psychological impact of quarantine is wide-ranging, substantial, and can be long-lasting [1]. Part of the impact can be seen as unpleasant feelings of isolation, loneliness, and boredom, which are different according to the people's susceptibility and previous experience. During regular periods, people who have easy access to safe and verdant outdoor space will feel more comfortable in public. [31].

Nearly all of the patients (n = 741, 96.86%) were emotionally stable before the outbreak. During the outbreak; however, about one-third of them (n = 521, 68.11%) became emotionally unstable because the social distancing and self-isolation had its impact on the emotional and psychological status of the concerned peoples. These effects are expressed by the patients as feelings of loneliness (n = 131, 17.12%), depression (n = 13, 01.69%), suicidal thoughts (00.13%), and overwhelming stress (n = 231, 30.19%).

The emotional impact continued after isolation calmed down, and there was a rise in overwhelming stress (n = 267, 34.90%), depression (n-148, 19.34%), suicidal thoughts (00.39%), and in contra, but after the end of the outbreak, there was a decrease in some emotional feelings like boredom (n = 76, 09.93%), and loneliness (n = 15, 01.96%). These feelings declined after the freeing from all types of quarantine among most patients (n = 509, 66.53).

The instability was in the form of feeling overwhelmingly stressed during the quarantine (n = 231, 30.19%), and after that period (n = 267, 34.90%). It is noticeable that this stress was present in more patients compared to the period of the outbreak. One hundred forty-five patients (18.95%) labeled themselves as bored during the outbreak, and a smaller number of them (n = 76, 09.93%) felt bored after the outbreak.

The same was true for a feeling of loneliness during (n = 131, 17.12%) and after (n = 15, 01.96%) the quarantine, respectively. However, depression was more after freedom from the blockade (n = 148, 19.34%) in comparison to the period of staying at home (n = 13, 01.69%). The same was true regarding suicidal thoughts; none of the patients ha this impulse before, while one patient (00.13%) developed it during the quarantine, and the number escalates to three patients after release from the blockade. This notion may be because the quarantine's impact was continuous after the period ended, and it was considered a factor most predictive of symptoms of acute stress disorder [1].

### 4.3 Gaining weight

Simply put, people gain weight when their calorie consumption from food intake exceeds the energy expenditure from physical activities [7], while sitting for a long time increases the risk of weight gain [8], and it may contribute to body weight gain [32,33].

The male patients (n = 138, 20.65%) outnumber the females (n = 73, 09.54%) in sustaining their previous weight after the outbreak, especially those from small cities and particularly from rural areas. This may be because running or any moderate or vigorous walking [9] or usual daily walk outside or even a picnic [31] in small groups were helpful in sustaining physical activities and their weight. In this regard, it has been stated that one could breathe safe and fresh air and increase those endorphins, decreasing stress and anxiety, and they feel more comfortable at least for a little while [9], and their mood will be boosted [12].

In rural areas with the already costumed family meals [2], absence of fast food [33], fewer hours of TV viewing, and lack of increased consumption of unhealthy foods, such as sugar-sweetened beverages, sweets [12, 33]. All these may have a positive effect on the struggle to maintain a healthy weight [20, 34].

As nutrition writer Bettina Elias Siegel points out, stress often increases the desire for highly palatable, unhealthy food [2]. During the COVID-19 pandemic filled with significant uncertainty, the exact method of staying on track with the weight loss goals might not be at the forefront of a mind [30].

It is now clear that emotional eating in stressed peoples is linked with gaining weight [24] due to emotion coping strategy [35]. Social isolation is cut across every aspect of human psychological and physiological functioning [29]. Regular gym customers have not enjoyed any fitness schedule at home [11]. There was a shortage of essential food groups, such as protein, grains, vegetables, and healthy fats, relying only on perishable items like canned goods [36]. All these with negative emotions contribute to weight gain [20]. Regarding weight gain, more males gained weight than females. A total of 410 patients (53.59%), including 239 males (31.24%) and171 females (22.35%), gained less than 1 kg.

Males had set a time for eating breakfast, lunch, and dinner, which may have limited any mindless snacking in between the meals. Eating breakfast [3,36, and 37] almost every day and avoiding eating between meals [38] reduce screen time and improve weight [3, 39]. Only eating in the kitchen or dining area and nowhere else in the house based on a little scheduling can go a long way during this stressful time [36].

The same was true for gaining 1–2 kg bodyweight as the males (n = 81, 10.58%) outnumbered the females (n = 48, 06.27%). While in gaining 3–5 kg, the females (n = 8, 01.45%) outnumbered the males (n = 4, 00.52%), and in gaining more than 5 kg, the females (n = 2, 00.26%) outnumbered the males (n = 1, 00.13%). The reason for this may be the fact that they were mostly from the center of the governorate and some small cities, while none of them were from rural areas. They were, in fact, staying at home all the time and have not set up a plan for their meals and snacks [11]. Moreover, sedentary behaviors were more prevalent among the females [39], and they were more prone to stress and worried about their and their families’ lives, expressing to be more socially stressed, which are positively correlated with gaining more weight [37] through various behavioral, psychological, and physiological pathways [28]. Increased energy intake and unhealthy food were chosen by women [39].

It is now clear that higher stress levels could contribute to obesity risk in women [10]. Also, other studies declared that physical activities are negatively correlated with gaining weight in females [8].

One of the limitations of the current study is related to its short period of recruitment of the patients. Another limitation is related to the fact that it was cross-sectional. Therefore, it is recommended that longitudinal studies should be carried out in the future.

## 5 Conclusions

Social distancing and self-isolation in the last COVID-19 outbreak influenced weight gain, but weight gain of less than 2 kg was observed among almost all patients who gained weight (98.05%). The patients who were gain more than 3 kg were mostly females or/and from the center of large cities.

## Recommendations

In tribal communities, tribal ceremonies such as sweat lodge, social gatherings, and seasonal ceremonies are a fundamental piece of social personality and usual and customary practices.

In the time of greater the danger of spreading the corona COVID-19, it is critical to make strides for the shirking of interest in tribal ceremonies, even funerals. This risk is particularly valid for those who might be higher, such as the elderly and people with underlying medical conditions.

Our brains are very social on so many levels; just having other people around us, even walking down the street, gives us a sense of security on an individual level. We experience things differently in the presence or absence of others.

We recommend that to allow small group picnic and outdoor activities in the fresh air are allowed provided that the no one, particularly the elders and people with underlying medical conditions, is at high risk and that has contact with corona-infected individuals, which will decrease the level of stress, emotional eating, and feelings of loneliness and sadness. These may not lead to gaining weight at the time of the breakout. However, precautions like staying at least six feet (or about two arms’ lengths) away from others, wearing masks, and not sharing or touching the same items are necessary.

## Conflicts of interest

Here to clarify and disclose any sources of any support for the work received in the form of grants and/or equipment and drugs.

To declare that there is no any conflict of interest.

## Figures and Tables

**Table:** 

The STROCSS Guideline		
Item no.	**Item description**	Page Number
**1**	Title. The words “cohort” and the area of focus should appear in the title (e.g. disease, exposure/intervention or outcome). Whether the study is retrospective or prospective should also be stated.	1, 5
**2a**	Abstract - Introduction What is the background and scientific rationale for the research question.	1
**2b**	Abstract - Methods - Describe the study design (cohort design, retrospective or prospective, single or multi-centre, etc), what was done to each group, how, when was it done and by whom.	1
**2c**	Abstract - Results What was found. Give the results for the main outcomes.	1
**2d**	Abstract - Conclusion - What have we learned and what does it mean. Where should future research go.	2
**3**	Explain the scientific background and rationale for the cohort study. What are objectives, research questions and the hypotheses.	3–4
**4a**	Registration and ethics State the research registry number in accordance with the declaration of Helsinki - "Every research study involving human subjects must be registered in a publicly accessible database before recruitment of the first subject" (this can be obtained from; ResearchRegistry.com or ClinicalTrials.gov or ISRCTN). Even retrospective studies should be registered prior to submission.	5
**4b**	Ethical Approval - State whether ethical approval was needed and if so, what the relevant judgement reference from the IRB or local ethics committee was? If ethical approval was not needed, state why.	5
**4c**	Protocol - Was a research protocol developed apriori? Where can it be accessed. Was it published in a journal e.g. IJS Protocols, BMJ Open, etc, if so, provide the reference.	5
**5a**	Study design - State the research is a cohort study and whether prospective or retrospective in design, whether single or multi-centre.	5
**5b**	Setting - Describe the setting(s)and nature of the institution in which the patient was managed; academic, community or private practice setting? Location(s), and relevant dates, including periods of recruitment, exposure, follow-up, and data collection	1
**5c**	Cohort Groups - State the number of groups in the study. What interventions will each group receive?	N/A
**5d**	Sub-group – Analysis. Any planned sub-group analyses are specified/Describe any methods used to examine subgroups and interactions.	
**6a**	Participants - State any eligibility (inclusion/exclusion) criteria and the sources and methods of selection of participants. Describe length and methods of follow-up.	5
**6b**	Recruitment - State the methods of how patients or participants were recruited to each group, over what time periods.	5
**6c**	Sample size calculation Whether there was calculation of margin of error or a prior analysis to determine study population, or mention of how appropriate study sample was determined.	5
**7a**	Pre-intervention considerations - e.g. Patient optimisation: measures taken prior to surgery or other intervention e.g. treating hypothermia/hypovolaemia/hypotension in burns patients, ICU care for sepsis, dealing with anticoagulation/other medications and so on.	5
**7b**	Types of intervention(s) deployed - To include reasoning behind treatment offered (pharmacological, surgical, physiotherapy, psychological, preventive) and concurrent treatments (antibiotics, analgesia, anti-emetics, nil by mouth, VTE prophylaxis, etc). Medical devices should have manufacturer and model specifically mentioned.	5
**7c**	Peri-intervention considerations - Administration of intervention (what, where, when and how was it done, including details for surgery; anaesthesia, patient position, use of tourniquet and other relevant equipment, preparation used, sutures, devices, surgical stage (1 or 2 stage, etc) and operative time. Pharmacological therapies should include formulation, dosage, strength, route and duration). Authors are encouraged to use figures, diagrams, photos, video and other multimedia to explain their intervention.	5
**7d**	Who performed the procedure(s) - Operator experience for each group (position on the learning curve for the technique if established, specialisation and prior relevant training).	5
**7e**	Quality control - What measures were taken to reduce inter or intra-operator variation. What measures were taken to ensure quality and consistency in the delivery of the intervention e.g. independent observers, lymph node counts, etc	5
**7f**	Post-intervention considerations - e.g. post-operative instructions and place of care. Important follow-up measures - diagnostic and other test results. Future surveillance requirements - e.g. imaging surveillance of endovascular aneurysm repair (EVAR) or clinical exam/ultrasound of regional lymph nodes for skin cancer.	5
**8**	Outcomes - What primary and secondary (if any) outcomes will be assessed and how are they defined. Definitions should be clear and precise. Appropriate references to validation of outcome measures used should be provided if they exist.	5
**9**	Statistical methods - Clearly outlined statistical tests used to compare the outcomes between an intervention group and a comparison group, state whether pre-existing differences and known confounders were controlled. The statistical package used should be mentioned.	5
**10a**	Participants recruited with a flow diagram - Report numbers involved in each group and use a flow diagram to show recruitment, non-participation, cross-over, withdrawal from the study with reasons.	5
**10b**	Comparison between groups including a table - Provide a table comparing the demographic, clinical/prognostic features (co-morbidities, tumour staging, smoking status, etc) and relevant socioeconomic characteristics of each group and whether numerical differences are significant (using p-values and/or confidence intervals as appropriate). Were the groups matched and if so, how.	N/A
**10c**	Changes - Any changes in the interventions during the course of the study (how has it evolved, been altered or tinkered with, what learning occurred, etc) together with rationale and a diagram if appropriate. Degree of novelty for a surgical technique/device should be mentioned and a comment on learning curves should be made for new techniques/devices.	N/A
**11a**	Outcomes and follow-up - Clinician assessed and patient-reported outcomes (when appropriate) should be stated for each group (size of effect with raw numbers and percentages) with inclusion of the time periods at which assessed. Relevant photographs/radiological images should be provided e.g. 12-month follow-up.Make it clear which confounders were adjusted for and which were not.	6
**11b**	Intervention adherence/compliance and tolerability - How was this assessed. Describe loss to follow-up (express as a percentage and a fraction) or cross-over between group and any explanations for them.	6
**11c**	Complications and adverse or unanticipated events - Described in detail and ideally categorised in accordance with the Clavien-Dindo Classification. How they were prevented, mitigated, diagnosed and managed. Blood loss, wound complications, re-exploration/revision surgery, 30-day post-op and long-term morbidity/mortality may need to be specified.	6
**12**	Summarise key results	6
**13**	Discussion of the relevance of the findings and rationale for conclusions - Relevant literature, implications for clinical practice guidelines, how have the indications for a new technique/device been refined and how do outcomes compare with established therapies and the prevailing gold standard should one exist and any relevant hypothesis generation. The rationale for any conclusions.	8
**14**	Strengths and limitations of the study	11
**15**	State what needs to be done next, further research with what study design(s).	
**16**	State the key conclusions from the study and key directions for future research	12
**17a**	State any conflicts of interest	14
**17b**	State any sources of funding	14

## Data Availability

The data are available on request through the author. Please contact the corresponding author professor Ahmed at hiwa.omer@univsul.edu.iq or 009647701530753 to request the data.

## Research registration unique identifying number (UIN)

Please enter the name of the registry and the unique identifying number of the study. You can register your research at http://www.researchregistry.com to obtain your UIN if you have not already registered your study. This is mandatory for human studies only.

Researchregistry5804

https://www.researchregistry.com/browse-the-registry#home/?view_2_search=researchregistry5804&view_2_page=1.
